# The Level of Adherence to Organic Food Consumption and Risk of Cancer: A Systematic Review and Meta-Analysis

**DOI:** 10.3390/life15020160

**Published:** 2025-01-23

**Authors:** Xenophon Theodoridis, Androniki Papaemmanouil, Niki Papageorgiou, Athina Vasiliki Georgakou, Ioustini Kalaitzopoulou, Marilena Stamouli, Michail Chourdakis

**Affiliations:** 1 Laboratory of Hygiene, Social and Preventive Medicine and Medical Statistics, School of Medicine, Faculty of Health Sciences, Aristotle University of Thessaloniki, 54124 Thessaloniki, Greece; xtheodoridis@auth.gr (X.T.); acpapaem@auth.gr (A.P.); npapaga@auth.gr (N.P.); georathi@auth.gr (A.V.G.); ioukalsav@auth.gr (I.K.); 2 Institute of Hepatology, Foundation for Liver Research, London SE5 9NT, UK; m.stamouli@researchinliver.org.uk; 3 School of Immunology and Microbial Sciences, Faculty of Life Sciences and Medicine, King’s College, London WC2R 2LS, UK

**Keywords:** tumor, organic food, frequency, systematic review

## Abstract

The available literature reports inconclusive findings regarding the frequency of organic food consumption and cancer incidence. This systematic review evaluated the effect of the frequency of organic food consumption on overall and site-specific cancer risk. Four electronic databases (PubMed, Scopus, Web of Science Core Collection, and Embase), the gray literature, and the reference lists of the included reports were searched for eligible studies. Study screening, data abstraction, and risk of bias assessment were performed by two independent examiners. Hazard ratios (HRs) and 95% confidence intervals using a random effects model were utilized to synthesize the available data from the included studies. There was no difference between the two interventions regarding overall cancer (HR = 0.93, 95% CI: 0.78–1.12), breast cancer (HR = 1.01, 95% CI: 0.81–1.26), colorectal cancer (HR = 1.01, 95% CI: 0.93–1.10), and non-Hodgkin lymphoma risks (HR = 0.70, 95% CI: 0.17–2.94). The findings suggest that the overall and site-specific cancer risk are not associated with the frequency of consumption of organic foods. Further research is necessary to provide more evidence for the role of organic food consumption on the incidence of cancer using homogeneous methodologies to define the frequency of organic food consumption.

## 1. Introduction

Worldwide, cancer is decreasing life expectancy and ranked at the top of the pyramid among the causes of human mortality [1]. Moreover, there is a rapid increase in cases and deaths due to cancer globally, which can be attributed to the growing number of the elderly population, the expansion of the global population, as well as the possible link to socioeconomic development and the prevalence of risk factors for cancer [2]. According to the Global Burden of Disease, 9.56 million people worldwide died from cancer in 2017. Cancer is believed to be one of the leading causes of death globally [3]. More specifically, in 2019, there were almost 23.6 million new cancer cases and 10 million deaths due to cancer [3]. With regard to breast cancer, there were 2.26 million new cases and 684.9 thousand deaths in 2020, whereas, for colon and rectum cancer, there were almost 2 million cases and 940 thousand deaths, respectively. For non-Hodgkin lymphoma, there were 544 thousand new incidences and 259 thousand deaths [4].

Dietary changes may lower the prevalence of cancer [5]. It is known that healthier nutritional choices, such as daily consumption of at least 400 g of fruits and vegetables, 30 g of fiber, whole-grain cereals, and pulses, may reduce the risk of developing cancer, as proposed by the World Cancer Research Fund and American Institute for Cancer Research [6]. Dietary patterns, including the Mediterranean diet, may prevent new cancer cases due to their anti-inflammatory and antioxidant components [7]. Additionally, specific food items have been recommended as potential anti-cancer interventions, including organic food. The study of organic food consumption as a treatment for cancer patients started early on in 1920 when Dr. Gerson used organic food as treatment, due to its possible therapeutical specialties [8]. The use of pesticides in conventional food agriculture is one of the main reasons why organic foods prevail, as they probably provide more beneficial components to humans than conventional food [9].

There is a plethora of definitions for organic food coming from the United States Department of Agriculture (USDA), the European Commission, and the Food and Agriculture Organization (FAO). According to the FAO definition, organic foods are produced and processed in accordance with special standards in order to qualify for the organic label [10]. Even though the increased consumption of organic foods may have a beneficial impact on humans’ health, more research is required in this new field [9]. There are several cohort studies that have examined the association between organic food consumption and health outcomes such as body weight change, risk of overweight or obesity [11,12], risk of pre-eclampsia [13,14], and the sociodemographic characteristics of the organic food consumers [15]. A previous systematic review focused on sustainable dietary patterns and cancer [16]. Of the seven studies that investigated the incidence of cancer, four examined exposure to pesticides or organic food consumption. According to the results, elevated organic food scores, indicating higher organic food consumption, were inversely associated with total cancer risk [16].

To our knowledge, there is no other systematic review that assesses the level of adherence to organic food consumption and the risk of cancer including all published studies. Thus, the aim of this systematic review and meta-analysis was to investigate the effect of the level of consumption (highest versus lowest consumption group) of organic foods on overall cancer and site-specific cancer risk.

## 2. Materials and Methods

This systematic review and meta-analysis protocol was submitted to the international registry of protocols for systematic reviews and meta-analyses, i.e., PROSPERO (CRD42023390668). The Reporting Guidelines for Meta-analyses of Observational Studies (MOOSE) [17] were followed for the present study. The MOOSE checklist is attached to the Supplementary Materials (Supplementary Table S1).

### 2.1. Search Strategy

A systematic search was performed using the electronic databases PubMed, Scopus, Web of Science Core Collection, and Embase for the inclusion of eligible studies, published in English, without a publication date restriction. The gray literature and the reference lists of the included studies were also searched for potentially eligible articles. The full search strategy is properly presented in the Supplementary Materials (Supplementary Table S2).

### 2.2. Eligibility Criteria

The PICO strategy was used to construct the research question and to define the eligibility criteria of this systematic review. The applied PICO process is presented in Table 1. Observational studies with an adult population were included in this systematic review. Comparisons between high and low consumption of organic foods, as well as studies reporting overall or site-specific cancer risk, were considered eligible for systematic review and meta-analysis. Studies performed on the pediatric population or those not reporting cancer risk were excluded.

### 2.3. Study Selection & Data Extraction

Data extraction was conducted independently by two reviewers. All retrieved references were imported into a reference management software (Rayyan, https://www.rayyan.ai/) [18]. After the removal of all duplicates, studies were screened for inclusion based on their titles and abstracts. The full-text screening was conducted for all included studies. Any disagreement was resolved by a third reviewer. The corresponding and first author of the original publication were contacted via email when study data were missing, unclear, or not reported in a form that could be used for the analysis.

For the eligible studies, a piloted data extraction Excel spreadsheet was used, which included study design, duration, follow-up period, type of population, participants, mean age (years), sex, organic food definition, mean energy intake (kcal/day), mean BMI (kg/m^2^), physical activity levels, smoking status, family history of cancer and menopausal hormone therapy.

### 2.4. Dietary Data Extraction

For this analysis of organic food consumption, findings from selected studies were drawn upon. Andersen et al. [19] categorized consumption into three levels—‘never’, ‘low’, and ‘high’—based on responses to a questionnaire that inquired about the consumption of six distinct food groups. These categories were established according to the score range, and quartiles were used for further classification. In the study conducted by Baudry et al. in 2018 [20], organic food consumption was assessed using data from three 24 h recall records. The National Nutrition Santé Guideline Score, excluding physical activity, was employed to calculate consumption levels. The population was subsequently divided into quartiles, ranging from the lowest to the highest consumption. In the research conducted by Bradbury et al. [21], participants were asked a simple question: ‘Do you consume organic food?’ They could choose from four possible responses: ‘never’, ‘sometimes’, ‘usually’, and ‘always’.

### 2.5. Quality Assessment

Each study’s risk of bias was assessed independently by two reviewers. Any disagreements were settled through discussion or by a third author. The ROBINS-E tool (Risk of Bias In Non-randomized Studies-of Exposures) [22] was used for the quality assessment of the eligible studies.

### 2.6. Data Synthesis

Hazard ratios (HRs) and 95% confidence intervals (CIs) were utilized to pool the effect size of each individual study using a random effects model. The Paule–Mandel method was used to estimate heterogeneity [23]. Statistical heterogeneity was quantified with τ^2^ and I^2^. Prediction intervals (PIs), a measure of reliability for the prediction of future observations, were also presented [24]. Publication bias could not be assessed due to the few included studies. All analyses were implemented in R Studio software (version 4.2.1) with the meta package.

### 2.7. Quality of the Evidence

The certainty of the evidence of our findings was assessed using the Grading of Recommendations, Assessment, Development, and Evaluations (GRADE) approach, as recommended by the *Cochrane Handbook* [25].

## 3. Results

### 3.1. Literature Search and Study Characteristics

**Figure 1:**
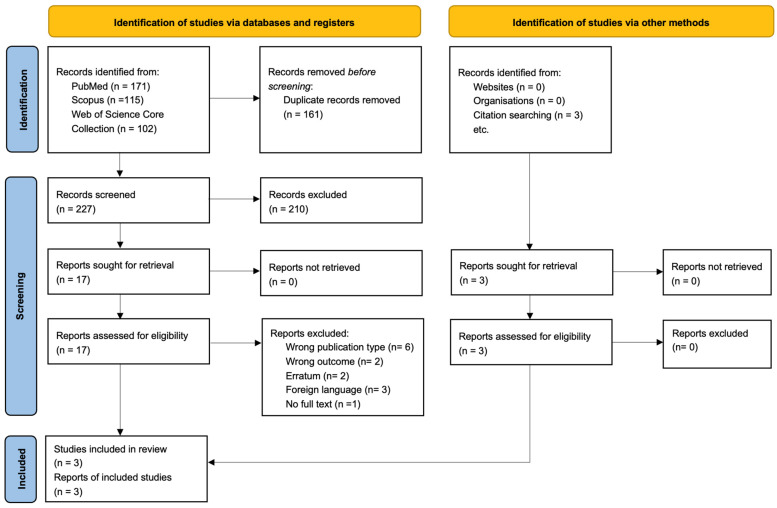
Flow diagram of the eligibility process.

Figure 1 shows the flow of the literature search and screening process. A total of 428 studies were identified based on a systematic search through PubMed, Scopus, Web of Science Core Collection, and Embase. After duplicate records were detected and then manually confirmed, 209 studies remained for further assessment. We screened them by title and abstract and proceeded to confirm eligibility for 17 studies according to our inclusion criteria. The final sample of the systematic review and meta-analysis included three observational studies.

The main characteristics of the included studies are summarized in Table 2. In summary, three cohort studies with a total of 733,954 individuals were included. Participants were adults with an average age of 54.5 years, and a predominance of females (80%).

### 3.2. Outcomes of Interest

#### 3.2.1. Overall Cancer

**Figure 2:**
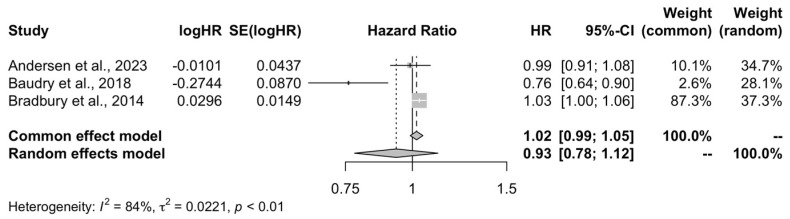
Meta-analysis forest plot of the high organic food consumption and the risk of overall cancer compared to low quartile of consumption [19,20,21].

A total of three studies [19,20,21] comprising 277,410 participants with 66,944 and 210,466 adults being in the group with the highest and lowest consumption of organic food, respectively, were included in the quantitative analysis to assess the association between organic food consumption and the risk of overall cancer. The results of the meta-analysis showed that high organic food consumption was not associated with the risk of overall cancer compared to the low quartile of consumption (HR = 0.93, 95% CI: 0.78–1.12, I^2^ = 84%, PI: 0.10–8.57) (Figure 2).

#### 3.2.2. Breast Cancer

**Figure 3:**
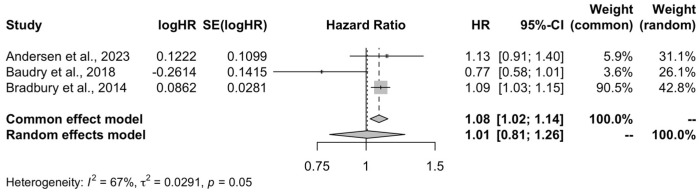
Meta-analysis forest plot of the high organic food consumption and the risk of breast cancer compared to low quartile of consumption [19,20,21].

The quantitative analysis included all three studies. The pooled HR in the included studies was 1.01 (95% CI: 0.81–1.26, I^2^ = 67%, PI: 0.07–13.55) (Figure 3).

#### 3.2.3. Colorectal Cancer

**Figure 4:**
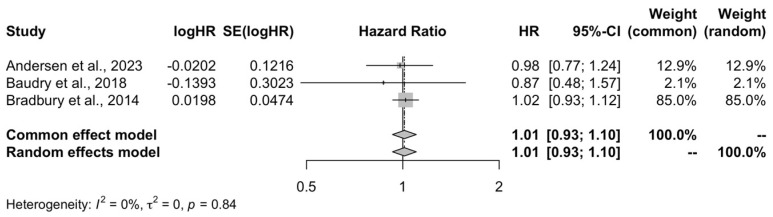
Meta-analysis forest plot of the high organic food consumption and the risk of colorectal cancer compared to low quartile of consumption [19,20,21].

The pooled estimate from the three included studies showed that there was no difference between high and low consumption of organic food regarding colorectal cancer risk (HR = 1.01, 95% CI: 0.93–1.10, I^2^ = 0%, PI: 0.58–1.76) (Figure 4).

#### 3.2.4. Non-Hodgkin Lymphoma

**Figure 5:**
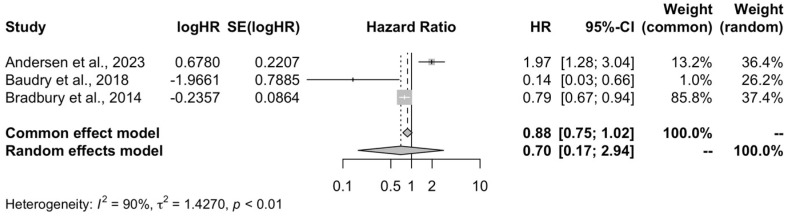
Meta-analysis forest plot of the high organic food consumption and the risk of non-Hodgkin cancer compared to low quartile of consumption [19,20,21].

High organic food consumption was not associated with the risk of non-Hodgkin lymphoma when compared to low consumption (HR = 0.70, 95% CI: 0.17–2.94, I^2^ = 90%) (Figure 5).

#### 3.2.5. Site-Specific Cancer

The results of the included studies regarding the effect of high consumption of organic foods compared to low consumption are presented in Table 3. Briefly, the study conducted by Baudry and colleagues [20] showed that high consumption of organic food is associated with a lower risk of postmenopausal breast cancer in comparison with the low consumption category. Furthermore, Bradbury and colleagues [21] demonstrated that a lower risk of non-Hodgkin lymphoma is related to higher organic food consumption. There was no difference in all the other site-specific cancers among the three included studies.

### 3.3. Risk of Bias Assessment

The results of the assessment of the risk of bias in the different domains of the ROBINS-E tool are presented in Supplementary Figures S1 and S2. Regarding the bias due to confounding, in all the included studies, some concerns were observed. Moreover, there were differences in the three included studies regarding the bias arising from the measurement of the exposure. In the study performed by Andersen et al. [19], there were some concerns; on the other hand, in the studies conducted by Baudry et al. [20] and Bradbury et al. [21], there was a high risk of bias due to the measurement of exposure. Most of the concerns about the studies were about the lack of definition of organic food and the fact that FFQ can lead to misclassification of exposure. Specifically, for the measurement of exposure, the study by Andresen et al. [19] provided more information about the exposure assessment rather than the studies of Baudry et al. [20] and Bradbury et al. [21]. Most of the rest of the domains of the tool were judged to be at low risk of bias for all included studies.

### 3.4. Certainty of Evidence

According to the GRADE approach, the certainty of the evidence for all of our endpoints was judged to be very low.

## 4. Discussion

This study is, to the best of our knowledge, the first to report the association between the highest and lowest consumption of organic foods using a systematic review and meta-analysis design. The findings indicate no difference between the two different levels of adherence to organic food consumption regarding the overall, breast, colorectal cancer, and non-Hodgkin lymphoma risk.

The association between organic food consumption and cancer risk remains uncertain, but there is evidence that factors like maintaining a healthy weight, staying physically active, and following a healthy diet may reduce cancer risk [26]. To prevent cancer, the American Cancer Society advises adopting a diet that minimizes red and processed meat, limits added sugars, replaces refined grains with whole grains, and includes more fruits and vegetables [27]. For overall health, the advantages of eating conventionally grown produce likely outweigh the potential risks from pesticide exposure [28]. Concerns about pesticides should not deter people from consuming conventional fruits and vegetables, particularly since organic options are often costly and not easily accessible to everyone [28].

The hypothesis that the consumption of organic foods leads to lower cancer incidence was generated based on the fact that organic foods do not contain chemicals originating from synthetic pesticides [9]. However, it should be noted that organic foods are not pesticide-free. Rather, they contain pesticides derived from natural substances. The reduction in synthetic pesticides in organic foods results in a decreased detection of urinary excretion of pesticides [20]. As long as individual pesticide concentrations in foods remain below the Maximum Residue Level, there is no evidence suggesting that dietary pesticide ingestion poses a health risk to consumers [9]. Notwithstanding, the International Agency for Research on Cancer (IARC) identified malathion and diazinon as probable carcinogenic pesticides to humans with a 2A classification and tetrachlorvinphos and parathion as possibly carcinogenic pesticides to humans with a 2B classification [29]. Among the identified mechanistic evidence for malathion and diazinon are genotoxicity and oxidative stress. Regarding malathion, inflammation, receptor-mediated effects, and cell proliferation or death have also been included as mechanistic evidence [29].

Pesticides have been a subject of concern regarding their potential impact on cancer development. Several studies have suggested a possible link between pesticide exposure and increased cancer risk [30,31,32,33]. Studies indicated that individuals exposed to pesticides had a higher likelihood of developing certain types of cancers, including leukemia and brain tumors [34,35]. The IARC, a specialized agency of the World Health Organization (WHO), has classified certain pesticides as probable or possible carcinogens based on available evidence. For example, the herbicide glyphosate, commonly used in agriculture, has been classified by the IARC as a probable carcinogen to humans (Group 2A) [36]. Glyphosate exposure has been linked to an increased risk of non-Hodgkin lymphoma in some studies [37]. Additionally, organophosphate pesticides, widely used in insect control, have been associated with various cancer types. A systematic review published in the *Reviews on Environmental Health* found a possible positive association between pesticides exposure and increased risk of prostate cancer [38]. Another study reported an elevated risk of childhood leukemia associated with parental occupational exposure to organophosphates [39].

In comparison to other nutritional epidemiology studies, evidence of how organic food consumption affects health outcomes in humans remains scarce [40]. Findings of different study designs assessing the relationship between organic food consumption and health outcomes have been evaluated by systematic reviews [40,41,42]. According to the findings of a systematic review [41], overall, there are no differences in selected biomarkers or nutrients between subjects consuming organic foods compared to participants consuming conventional foods. The results of these systematic reviews are in agreement with studies evaluating the antioxidant capacity, nutrients in biomarkers, or nutrition-related health outcomes [42]. Even though organically produced foods have greater concentrations of vitamins, minerals, and beneficial fatty acids compared to conventionally produced food, these compositional differences are unlikely to have an effect on populations living in areas where nutrient availability is adequate [43].

On the other hand, observational studies assessing the effect of organic food consumption compared to the conventional one or the level of adherence to organic food consumption have many confounding factors. For example, the data show that organic food consumers exhibit healthy dietary patterns such as consuming plant-based foods and limiting sugary and alcoholic beverages, processed meat, and milk [44]. They also tend to present a higher compliance with dietary guidelines [45] and be more physically active [9]. A recently published systematic review and meta-analysis showed that participants consuming organic foods had a lower probability of being classified in the overweight or obesity category in comparison with the unexposed group [12], another important confounder linked to cancer risk [46,47]. Thus, disentangling the direct effect of organic food consumption from these confounders remains challenging.

Among the strengths of our study is the adherence to the Cochrane guidelines and the use of sound methodology to retrieve and synthesize the available evidence. We acknowledge that the inclusion of English-only published studies is among our limitations. However, there are published meta-epidemiologic studies showing that restricting systematic reviews to English-language publications appears to have little or minimal impact on the effect estimates and conclusions of systematic reviews [48,49].

There are also several limitations that should be addressed in the assessment of the frequency of organic food consumption and the possible impact on cancer risk. The assessment of food consumption, in general, can be biased due to the self-reported nature of the food frequency questionnaires that participants must complete [50]. Participants tend to over-report their healthy food choices while, on the contrary, they under-report more energy-dense and less nutrient-dense foods. Furthermore, the variability in portion sizes, cooking methods, and the preparation of each meal may be considered as a possible limitation and thus impact the accuracy of the food frequency questionnaire [50].

Moreover, there is substantial heterogeneity in the definition and labeling of organic food products. There is a chance that participants’ perceptions of organic food will differ depending on regional regulatory requirements and labeling changes throughout time. There are different definitions regarding organic food, depending on the organization of each continent. More specifically, the USDA defines organic foods as food items produced by farmers without using most of the conventional pesticides, synthetic fertilizers, sewage sludge, or genetically modified organisms (GMOs) while in parallel emphasizing resource cycling [51]. In the European Union, the European Commission has labeled organic foods as those that have been produced by organic farms and that promote specific agricultural practices, in terms of respecting the environment and animals. Also, those farms should not use synthetic pesticides and fertilizers [52]. These misunderstandings may lead to misclassification bias, which may skew the association between the reported use of organic foods and the risk of developing cancer.

Residual confounding can also be considered as a limitation. Relevant factors apart from age, sex, and socioeconomic status can be unmeasured or not properly adjusted in epidemiologic studies, such as dietary behaviors, physical activity, or medical history [53]. These factors may influence the consumption of organic food but also the risk of developing cancer.

The possibility of self-selection bias should also be addressed. Participants who volunteer to participate in dietary research may be more health-conscious than the general population [54], making them more inclined to adopt organic eating patterns. Because of this selection bias, the genuine connection between organic food consumption frequency and cancer risk may be overestimated. Future studies should involve *a priori* screening for diet quality in order to better describe potential participants prior to trial enrolment [55]. Furthermore, the use of biochemical markers related to organic food consumption is essential to ensuring the baseline and follow-up status of pesticide levels in urine, a potential marker of organic food consumption [55,56].

Finally, there are only a few observational studies that have studied the association between organic food consumption and the risk of developing cancer. Hence, the results of this systematic review should be interpreted with caution. Future studies with further research on this topic should be addressed, using an accepted definition of organic food, and a possible questionnaire for organic food, in order to have the same way of measuring the quantity of food.

To summarize, assessing the frequency of organic food consumption in relation to cancer risk is a complicated task with numerous limitations. Measurement errors, changes in organic definitions, residual confounding, reporting biases, and potential selection biases are all examples of these. Moreover, the lack of homogeneity in methodologies was a limitation for valid comparisons, and a similar methodology in all studies would be helpful and welcomed in future studies. Recognizing and overcoming these limitations is critical for correctly interpreting study findings and directing future research practices in this field.

## 5. Conclusions

These findings suggest that the overall and site-specific cancer risks are not associated with the frequency of consumption of organic foods. More research is necessary to provide more evidence for the role of organic food consumption in the incidence of cancer using homogeneous methodologies to define the frequency of organic food consumption and make valid comparisons.

## Figures and Tables

**Table 1:** Description of the PICO strategy.

PICO Acronym Criteria	PICO Items Relevant to Eligibility Criteria
P—Population	Adult population without a cancer diagnosis
I—Intervention	High consumption of organic foods
C—Control	Low or no consumption of organic foods
O—Outcome	Overall and site-specific cancer risk
S—Study Design	Observational studies

**Table 2:** Study characteristics of the included studies.

First Author, Year, Country	Sample Size ^1^	Population	Exposure Assessment	Outcome Assessment	Follow-Up (Mean Years)	Covariates
Andersen et al., 2023 [19], Denmark	Total: 41,928 Never: 6184 Low: 16,246 Medium: 15,374 High: 4124	Danish adults aged 50–65 years with no previous cancer diagnosis	Follow-up FFQ (questions about the consumption of or organically produced foods) and a follow-up lifestyle questionnaire.	Linkage with the Danish Cancer Registry	15	Educational level, BMI, PA, smoking habits
Baudry et al., 2018 [20], France	Total: 68,946 Q1: 16,831 Q2: 17,644 Q3: 17,240 Q4: 17,231	French adult volunteers with access to the internet	Self-administrated questionnaires, 24 h records, and anthropometric questionnaires.	Self-reported health events through yearly health status questionnaire	4.56	Age, sex, month of inclusion, occupational status, educational level, marital status, monthly income per household unit, PA, smoking status, alcohol intake, family history of cancer, BMI, height, energy intake, mPNNS-GS, fiber intake, processed meat intake, red meat intake, ultra-processed food consumption, fruit and vegetable consumption, dietary patterns, and (for women) parity, postmenopausal status, use of hormonal treatment for menopause, and use of oral contraception
Bradbury et al., 2014 [21], United Kingdom	Total: 623,080 Never: 187,451 Sometimes: 390,040 Usually/Always: 45,589	Middle-aged women who had been invited for breast cancer screening at screening centers	At the 3-year survey, women were asked ‘Do you eat organic food?’ with four possible categorical responses. Women were also asked if they had changed their diet because of illness in the previous 5 years. The same questions were asked in the 8-year survey.	Participants are flagged on the NHSCR, so that cancer registrations and deaths are routinely notified to the study investigators by the Office for National Statistics, England, and the Information Services Division, Scotland	9.3	Age, geographical region, socioeconomic status, BMI, height, smoking status, alcohol intake, strenuous PA, age at first birth, fiber intake, type of meat eaten

BMI: Body Mass Index; FFQ: food frequency questionnaire; mPNNS-GS: Modified Version of the Validated Programme National Nutrition Santé Guideline Score without the physical activity component; NHSCR: National Health Service Central Register; PA: physical activity. ^1^ Sample size refers to the total study population.

**Table 3:** Results of the included studies regarding the effect of organic food consumption.

	Andersen et al., 2023 ^1^ [19]	Baudry et al., 2018 ^2^ [20]	Bradbury et al., 2014 ^3^ [21]
Overall cancer	0.99 (0.91–1.08)	0.76 (0.64–0.90)	1.03 (1.00–1.06)
Pancreas cancer	1.00 (0.61–1.65)		1.06 (0.87–1.29)
Lung cancer	0.79 (0.60–1.04)		0.98 (0.88–1.10)
Stomach cancer	0.54 (0.27–1.07)		0.92 (0.67–1.26)
Colorectal cancer	0.98 (0.77–1.24)	0.87 (0.45–1.57)	1.02 (0.93–1.12)
Urinary bladder cancer	0.91 (0.62–1.31)		
Non-Hodgkin lymphoma	1.97 (1.28–3.04)	0.14 (0.03–1.05)	0.79 (0.67–0.94)
Breast cancer	1.13 (0.91–1.40)	0.77 (0.58–1.01)	1.09 (1.03–1.15)
Premenopausal breast cancer		0.89 (0.59–1.35)	
Postmenopausal breast cancer		0.66 (0.45–0.96)	
Prostate cancer	0.95 (0.78–1.15)	1.00 (0.63–1.60)	
Skin cancer		0.63 (0.38–1.05)	
Kidney cancer			0.83 (0.64–1.08)
Brain cancer			1.16 (0.97–1.40)
Leukemia			0.92 (0.72–1.18)
Malignant melanoma			0.90 (0.78–1.05)
Esophagus			0.83 (0.62–1.12)
Oral cavity			1.04 (0.78–1.39)
Uterus			1.14 (1.00–1.29)
Ovary			0.96 (0.83–1.11)
Bladder cancer			1.08 (0.82–1.41)
Multiple myeloma			1.03 (0.81–1.32)

^1^ high vs. never. ^2^ high vs. low. ^3^ usually/always vs. never.

## Data Availability

The data presented in this study are available in the main text and Supplementary Materials.

## Supplementary Materials

The following supporting information can be downloaded at https://www.mdpi.com/article/10.3390/life15020160/s1, Table S1: MOOSE checklist for meta-analyses of observational studies; Table S2: Search strategy for identifying observational studies on PubMed; Figure S1: Risk of bias assessment presented as traffic lights; Figure S2: Risk of bias assessment presented as bar plots.

## References

[1] World Health Organization: Regional Office For Europe (2020). World Cancer Report: Cancer Research for Cancer Development.

[2] Sung H., Ferlay J., Siegel R.L., Laversanne M., Soerjomataram I., Jemal A., Bray F. (2021). Global Cancer Statistics 2020: GLOBOCAN Estimates of Incidence and Mortality Worldwide for 36 Cancers in 185 Countries. CA Cancer J. Clin..

[3] Global Burden of Disease 2019 Cancer Collaboration (2022). Cancer Incidence, Mortality, Years of Life Lost, Years Lived with Disability, and Disability-Adjusted Life Years for 29 Cancer Groups from 2010 to 2019: A Systematic Analysis for the Global Burden of Disease Study 2019. JAMA Oncol..

[4] Ferlay J., Ervik M., Lam F., Colombet M., Mery L., Piñeros M., Znaor A., Soerjomataram I., Bray F. (2020). Global Cancer Observatory: Cancer Today.

[5] Donaldson M.S. (2004). Nutrition and Cancer: A Review of the Evidence for an Anti-Cancer Diet. Nutr. J..

[6] World Cancer Research Fund, American Institute for Cancer Research (2018). Diet, Nutrition, Physical Activity and Cancer: A Global Perspective. Continuous Update Project Expert Report.

[7] Mentella M.C., Scaldaferri F., Ricci C., Gasbarrini A., Miggiano G.A.D. (2019). Cancer and Mediterranean Diet: A Review. Nutrients.

[8] Bishop B. (1988). Organic Food in Cancer Therapy. Nutr. Health.

[9] Vigar V., Myers S., Oliver C., Arellano J., Robinson S., Leifert C. (2020). A Systematic Review of Organic versus Conventional Food Consumption: Is There a Measurable Benefit on Human Health?. Nutrients.

[10] Food and Agriculture Organization of the United Nations Organic Agriculture. https://openknowledge.fao.org/server/api/core/bitstreams/b5377c27-d4b1-4cf1-9186-3eb7e702a18b/content.

[11] Kesse-Guyot E., Baudry J., Assmann K.E., Galan P., Hercberg S., Lairon D. (2017). Prospective Association between Consumption Frequency of Organic Food and Body Weight Change, Risk of Overweight or Obesity: Results from the NutriNet-Santé Study. Br. J. Nutr..

[12] Bhagavathula A.S., Vidyasagar K., Khubchandani J. (2022). Organic Food Consumption and Risk of Obesity: A Systematic Review and Meta-Analysis. Healthcare.

[13] Torjusen H., Brantsæter A.L., Haugen M., Alexander J., Bakketeig L.S., Lieblein G., Stigum H., Næs T., Swartz J., Holmboe-Ottesen G. (2014). Reduced Risk of Pre-Eclampsia with Organic Vegetable Consumption: Results from the Prospective Norwegian Mother and Child Cohort Study. BMJ Open.

[14] Simões-Wüst A.P., Moltó-Puigmartí C., Jansen E.H.J.M., Van Dongen M.C.J.M., Dagnelie P.C., Thijs C. (2017). Organic Food Consumption during Pregnancy and Its Association with Health-Related Characteristics: The KOALA Birth Cohort Study. Public Health Nutr..

[15] Petersen S.B., Rasmussen M.A., Strom M., Halldorsson T.I., Olsen S.F. (2013). Sociodemographic Characteristics and Food Habits of Organic Consumers—A Study from the Danish National Birth Cohort. Public Health Nutr..

[16] Karavasiloglou N., Pannen S.T., Jochem C., Kuhn T., Rohrmann S. (2022). Sustainable Diets and Cancer: A Systematic Review. Curr. Nutr. Rep..

[17] Brooke B.S., Schwartz T.A., Pawlik T.M. (2021). MOOSE Reporting Guidelines for Meta-Analyses of Observational Studies. JAMA Surg..

[18] Ouzzani M., Hammady H., Fedorowicz Z., Elmagarmid A. (2016). Rayyan-a Web and Mobile App for Systematic Reviews. Syst. Rev..

[19] Andersen J.L.M., Frederiksen K., Hansen J., Kyrø C., Overvad K., Tjønneland A., Olsen A., Raaschou-Nielsen O. (2023). Organic Food Consumption and the Incidence of Cancer in the Danish Diet, Cancer and Health Cohort. Eur. J. Epidemiol..

[20] Baudry J., Assmann K.E., Touvier M., Allès B., Seconda L., Latino-Martel P., Ezzedine K., Galan P., Hercberg S., Lairon D. (2018). Association of Frequency of Organic Food Consumption With Cancer Risk: Findings From the NutriNet-Santé Prospective Cohort Study. JAMA Intern. Med..

[21] Bradbury K.E., Balkwill A., Spencer E.A., Roddam A.W., Reeves G.K., Green J., Key T.J., Pirie K., Banks E., Beral V. (2014). Organic Food Consumption and the Incidence of Cancer in a Large Prospective Study of Women in the United Kingdom. Br. J. Cancer.

[22] ROBINS-E Development Group Risk of Bias in Non-Randomized Studies—Of Exposure (ROBINS-E). https://pubmed.ncbi.nlm.nih.gov/38555664/.

[23] Veroniki A.A., Jackson D., Viechtbauer W., Bender R., Bowden J., Knapp G., Kuss O., Higgins J.P., Langan D., Salanti G. (2016). Methods to Estimate the Between-Study Variance and Its Uncertainty in Meta-Analysis. Res. Synth. Methods.

[24] Ramachandran K.M., Tsokos C.P., Ramachandran K.M., Tsokos C.P. (2021). Chapter 14—Some Issues in Statistical Applications: An Overview. Mathematical Statistics with Applications in R.

[25] Higgins J.P.T., Thomas J., Chandler J., Cumpston M., Li T., Page M.J., Welch V.A. (2019). Cochrane Handbook for Systematic Reviews of Interventions.

[26] Katzke V.A., Kaaks R., Kühn T. (2015). Lifestyle and Cancer Risk. Cancer J..

[27] Rock C.L., Thomson C., Gansler T., Gapstur S.M., McCullough M.L., Patel A.V., Andrews K.S., Bandera E.V., Spees C.K., Robien K. (2020). American Cancer Society Guideline for Diet and Physical Activity for Cancer Prevention. CA Cancer J. Clin..

[28] Hemler E.C., Chavarro J.E., Hu F.B. (2018). Organic Foods for Cancer Prevention-Worth the Investment?. JAMA Intern. Med..

[29] Guyton K.Z., Loomis D., Grosse Y., el Ghissassi F., Benbrahim-Tallaa L., Guha N., Scoccianti C., Mattock H., Straif K., Blair A. (2015). Carcinogenicity of Tetrachlorvinphos, Parathion, Malathion, Diazinon, and Glyphosate. Lancet Oncol..

[30] Alavanja M.C.R., Ross M.K., Bonner M.R. (2013). Increased Cancer Burden among Pesticide Applicators and Others Due to Pesticide Exposure. CA Cancer J. Clin..

[31] Maele-Fabry G.V., Willems J.L. (2003). Occupation Related Pesticide Exposure and Cancer of the Prostate: A Meta-Analysis. Occup. Environ. Med..

[32] Pardo L.A., Beane Freeman L.E., Lerro C.C., Andreotti G., Hofmann J.N., Parks C.G., Sandler D.P., Lubin J.H., Blair A., Koutros S. (2020). Pesticide Exposure and Risk of Aggressive Prostate Cancer among Private Pesticide Applicators. Environ. Health.

[33] Panis C., Candiotto L.Z.P., Gaboardi S.C., Gurzenda S., Cruz J., Castro M., Lemos B. (2022). Widespread Pesticide Contamination of Drinking Water and Impact on Cancer Risk in Brazil. Environ. Int..

[34] Schinasi L., Leon M.E. (2014). Non-Hodgkin Lymphoma and Occupational Exposure to Agricultural Pesticide Chemical Groups and Active Ingredients: A Systematic Review and Meta-Analysis. Int. J. Environ. Res. Public Health.

[35] Gatto N.M., Ogata P., Lytle B. (2021). Farming, Pesticides, and Brain Cancer: A 20-Year Updated Systematic Literature Review and Meta-Analysis. Cancers.

[36] International Agency for Research on Cancer (IARC) (2017). Some Organophosphate Insecticides and Herbicides Volume 112 Iarc Monographs on the Evaluation of Carcinogenic Risks to Humans.

[37] Zhang L., Rana I., Shaffer R.M., Taioli E., Sheppard L. (2019). Exposure to glyphosate-based herbicides and risk for non-Hodgkin lymphoma: A meta-analysis and supporting evidence. Mutat. Res. Rev. Mutat. Res..

[38] Silva J.F.S., Mattos I.E., Luz L.L., Carmo C.N., Aydos R.D. (2016). Exposure to pesticides and prostate cancer: Systematic review of the literature. Rev. Environ. Health.

[39] Turner M.C., Wigle D.T., Krewski D. (2010). Residential Pesticides and Childhood Leukemia: A Systematic Review and Meta-Analysis. Environ. Health Perspect..

[40] Mie A., Andersen H.R., Gunnarsson S., Kahl J., Kesse-Guyot E., Rembiałkowska E., Quaglio G., Grandjean P. (2017). Human Health Implications of Organic Food and Organic Agriculture: A Comprehensive Review. Environ. Health.

[41] Smith-Spangler C., Brandeau M.L., Hunter G.E., Clay Bavinger J., Pearson M., Eschbach P.J., Sundaram V., Liu H., Schirmer P., Stave C. (2012). Are Organic Foods Safer or Healthier than Conventional Alternatives?: A Systematic Review. Ann. Intern. Med..

[42] Dangour A.D., Lock K., Hayter A., Aikenhead A., Allen E., Uauy R. (2010). Nutrition-Related Health Effects of Organic Foods: A Systematic Review. Am. J. Clin. Nutr..

[43] Brantsæter A.L., Ydersbond T.A., Hoppin J.A., Haugen M., Meltzer H.M. (2017). Organic Food in the Diet: Exposure and Health Implications. Annu. Rev. Public Health.

[44] Kesse-Guyot E., Péneau S., Méjean C., Szabo de Edelenyi F., Galan P., Hercberg S., Lairon D. (2013). Profiles of Organic Food Consumers in a Large Sample of French Adults: Results from the Nutrinet-Santé Cohort Study. PLoS ONE.

[45] Baudry J., Méjean C., Péneau S., Galan P., Hercberg S., Lairon D., Kesse-Guyot E. (2015). Health and Dietary Traits of Organic Food Consumers: Results from the NutriNet-Santé Study. Br. J. Nutr..

[46] Mctiernan A., Friedenreich C.M., Katzmarzyk P.T., Powell K.E., Macko R., Buchner D., Pescatello L.S., Bloodgood B., Tennant B., Vaux-Bjerke A. (2019). Physical Activity in Cancer Prevention and Survival: A Systematic Review. Med. Sci. Sports Exerc..

[47] Freisling H., Arnold M., Soerjomataram I., O’Doherty M.G., Ordóñez-Mena J.M., Bamia C., Kampman E., Leitzmann M., Romieu I., Kee F. (2017). Comparison of General Obesity and Measures of Body Fat Distribution in Older Adults in Relation to Cancer Risk: Meta-Analysis of Individual Participant Data of Seven Prospective Cohorts in Europe. Br. J. Cancer.

[48] Nussbaumer-Streit B., Klerings I., Dobrescu A.I., Persad E., Stevens A., Garritty C., Kamel C., Affengruber L., King V.J., Gartlehner G. (2020). Excluding Non-English Publications from Evidence-Syntheses Did Not Change Conclusions: A Meta-Epidemiological Study. J. Clin. Epidemiol..

[49] Dobrescu A.I., Nussbaumer-Streit B., Klerings I., Wagner G., Persad E., Sommer I., Herkner H., Gartlehner G. (2021). Restricting Evidence Syntheses of Interventions to English-Language Publications Is a Viable Methodological Shortcut for Most Medical Topics: A Systematic Review. J. Clin. Epidemiol..

[50] Shim J.-S., Oh K., Kim H.C. (2014). Dietary Assessment Methods in Epidemiologic Studies. Epidemiol. Health.

[51] U.S. Department of Agriculture, A.M.S Labeling Organic Products. https://www.ams.usda.gov/rules-regulations/organic/labeling.

[52] Agriculture and Rural Development Organic Production and Products. https://agriculture.ec.europa.eu/farming/organic-farming/organic-production-and-products_en.

[53] Fewell Z., Davey Smith G., Sterne J.A.C. (2007). The Impact of Residual and Unmeasured Confounding in Epidemiologic Studies: A Simulation Study. Am. J. Epidemiol..

[54] Young L.M., Gauci S., Scholey A., White D.J., Pipingas A. (2020). Self-Selection Bias: An Essential Design Consideration for Nutrition Trials in Healthy Populations. Front. Nutr..

[55] Heaney R.P. (2014). Guidelines for Optimizing Design and Analysis of Clinical Studies of Nutrient Effects. Nutr. Rev..

[56] Hyland C., Bradman A., Gerona R., Patton S., Zakharevich I., Gunier R.B., Klein K. (2019). Organic Diet Intervention Significantly Reduces Urinary Pesticide Levels in U.S. Children and Adults. Environ. Res..

