# Supplementary material for: The Level of Adherence to Organic Food Consumption and Risk of Cancer: A Systematic Review and Meta-Analysis
Source: Life (Basel). 2025 Jan 23;15(2):160. doi: 10.3390/life15020160 (PMC11856173; doi:10.3390/life15020160)

Contents

Supplementary Table S1: MOOSE Checklist for Meta-analyses of Observational Studies

Supplementary Table S2: Search strategy for identifying observational studies on PubMed

Supplementary Figure S1: Risk of bias assessment presented as traffic lights

Supplementary Figure S2: Risk of bias assessment presented as bar plots

Table S1: MOOSE Checklist for Meta-analyses of Observational Studies

Item No	Recommendation	Reported on Page No
Reporting of background should include		
1	Problem definition	1
2	Hypothesis statement	1-2
3	Description of study outcome(s)	3
4	Type of exposure or intervention used	3
5	Type of study designs used	3
6	Study population	3
Reporting of search strategy should include		
7	Qualifications of searchers (eg, librarians and investigators)	2
8	Search strategy, including time period included in the synthesis and key words	2, Supplementary Table S2
9	Effort to include all available studies, including contact with authors	3
10	Databases and registries searched	2
11	Search software used, name and version, including special features used (eg, explosion)	2
12	Use of hand searching (eg, reference lists of obtained articles)	2
13	List of citations located and those excluded, including justification	Figure 1
14	Method of addressing articles published in languages other than English	-
15	Method of handling abstracts and unpublished studies	2-3
16	Description of any contact with authors	3
Reporting of methods should include		
17	Description of relevance or appropriateness of studies assembled for assessing the hypothesis to be tested	3
18	Rationale for the selection and coding of data (eg, sound clinical principles or convenience)	3
19	Documentation of how data were classified and coded (eg, multiple raters, blinding and interrater reliability)	-
20	Assessment of confounding (eg, comparability of cases and controls in studies where appropriate)	-
21	Assessment of study quality, including blinding of quality assessors, stratification or regression on possible predictors of study results	3
22	Assessment of heterogeneity	4
23	Description of statistical methods (eg, complete description of fixed or random effects models, justification of whether the chosen models account for predictors of study results, dose-response models, or cumulative meta-analysis) in sufficient detail to be replicated	4
24	Provision of appropriate tables and graphics	Figure 1, Tables 2-3, Figures 2-5
Reporting of results should include		

25	Graphic summarizing individual study estimates and overall estimate	Figures 2-5, Table 3
26	Table giving descriptive information for each study included	Table 2
27	Results of sensitivity testing (eg, subgroup analysis)	-
28	Indication of statistical uncertainty of findings	9

Item No	Recommendation	Reported on Page No
Reporting of discussion should include		
29	Quantitative assessment of bias (eg, publication bias)	N/A
30	Justification for exclusion (eg, exclusion of non-English language citations)	11
31	Assessment of quality of included studies	9, Supplementary Figures S1-S2
Reporting of conclusions should include		
32	Consideration of alternative explanations for observed results	9-12
33	Generalization of the conclusions (ie, appropriate for the data presented and within the domain of the literature review)	-
34	Guidelines for future research	12
35	Disclosure of funding source	12

Table S2: Search strategy for identifying observational studies on PubMed.

Search	Query
#1	Organic food [MeSH Terms]
#2	Organic foods [MeSH Terms]
#3	Organic food* [Title/Abstract]
#4	#1 – #3/OR
#5	Cancer [MeSH Terms]
#6	Cancer [Title/Abstract]
#7	Tumor [Title/Abstract]
#8	Tumor [Title/Abstract]
#9	Neoplasm [Title/Abstract]
#10	Neoplasm* [Title/Abstract]
#11	Malignant [Title/Abstract]
#12	Carcinoma [Title/Abstract]
#13	Carcinoma* [Title/Abstract]
#14	Malignant neoplasia [Title/Abstract]
#15	Malignant neoplasm* [Title/Abstract]
#16	Malignant neoplastic disease [Title/Abstract]
#17	Malignant tumor* [Title/Abstract]
#18	Malignant tumor* [Title/Abstract]
#19	Neoplasmia [Title/Abstract]
#20	#5 – #19/OR
#21	#4 AND #20
The last search was performed on 6th April 2024	

Supplementary Figure S1: Risk of bias assessment presented as traffic lights.

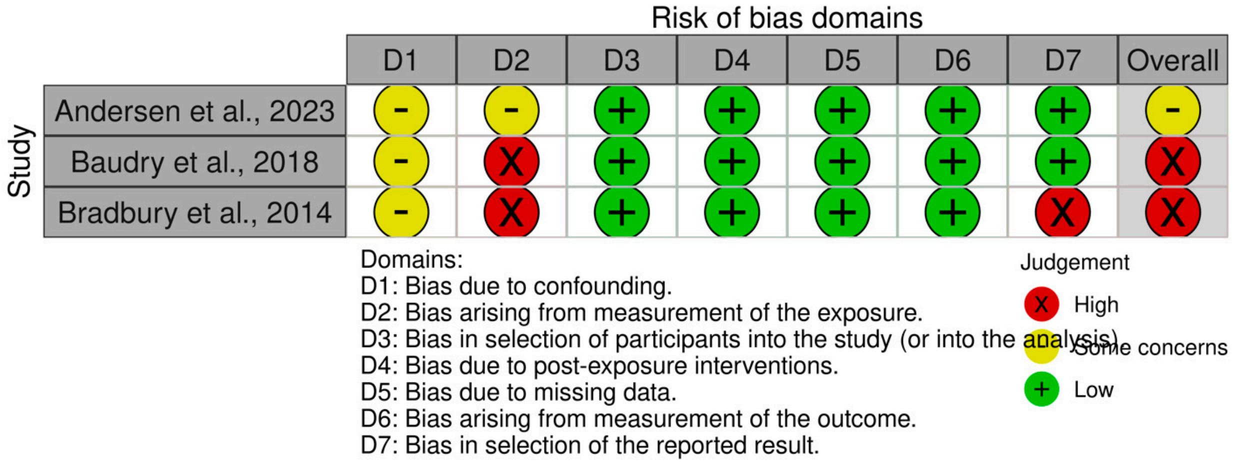

Supplementary Figure S2: Risk of bias assessment presented as bar plots.

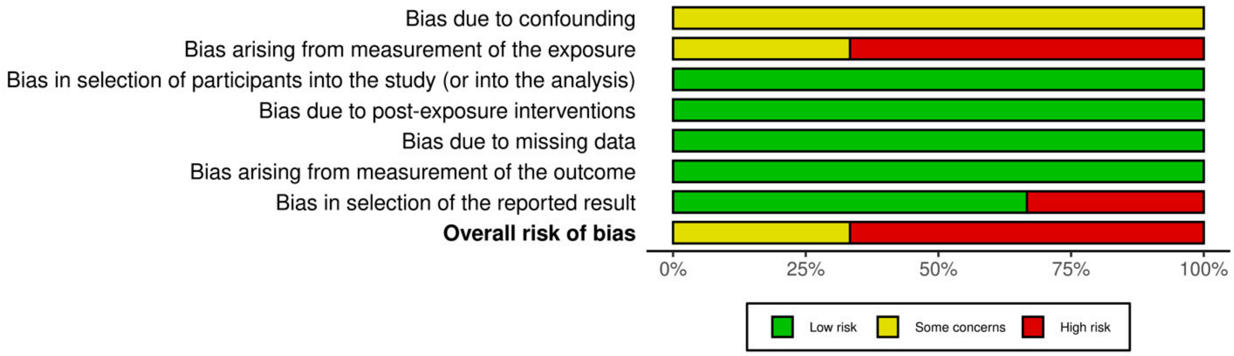

Supplement: Supplementary file 1 [file life-15-00160-s001.zip › life-3343465-supplementary.pdf]
